# Do housing prices affect individual physical health? Evidence from China

**DOI:** 10.1371/journal.pone.0299561

**Published:** 2024-04-17

**Authors:** Rui Zhang

**Affiliations:** School of International Trade and Economics, University of International Business and Economics, Beijing, China; Lingnan University, The University of HongKong, HONG KONG

## Abstract

This study identifies the health effect of rising housing prices on individual physical health using the Chinese General Social Survey (CGSS) data. Exploiting exogenous housing prices, I find that rising housing prices adversely affect physical health status. Heterogeneity analyses yield interesting findings. First, the adverse effects of high housing prices are pronounced in the group owning only one house. Second, significant effects of housing prices on health for the group aged 20 to 45 are observed, with no effects for the elderly group above 45. Third, males are more sensitive to high housing prices due to the intensified competition and traditional gender norm in marriage markets. I also further investigate the channel through which housing prices affect individual physical health. The findings indicate that rising housing prices can damage individual physical health via lowering social status, reducing physical exercise time and increasing mental health risk.

## Section 1: Introduction

China’s health issues are receiving public attention with the country’s economic development and social progress. In 2009, the Chinese Government launched “Healthy China 2020”. The program has been consolidated and extended, known as the “Healthy China 2030”, indicating that public health issues have become a national development strategy. With many efforts in improving public health, China has made substantial progress. However, accompanied by urbanization, an increasing number of Chinese are stuck in suboptimal health status because of pressure, depression, fatigue, and low self-esteem emotions induced by buying a house. The 2012 Urban Residents Health White Paper Survey, which is released by Chinese Medical Doctor Association, reports that rising housing prices ranks as the top threats to health.

**Fig 1:**
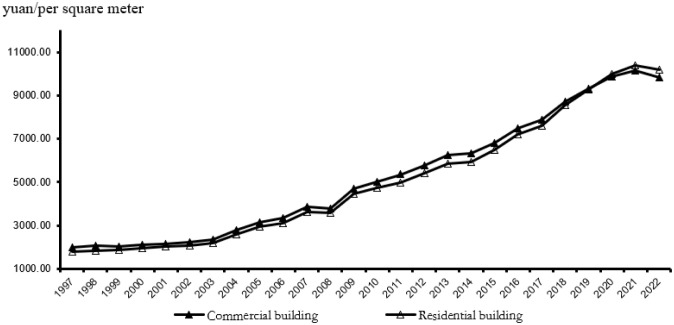
National average housing prices. The data come from the China Statistical Yearbooks.

Notably, real estate prices have risen after the housing reform in 1998. As Fig 1 shows, housing prices have increased more rapidly since 2003. To handle the 2008 financial crisis, the Chinese government announced a 4 trillion-yuan stimulus plan to spur the economic, which again pushed up the housing prices. According to the National Bureau of Statistics of China, the national average sales prices of residential house increased by two times from 2008 to 2021. Despite the tremendous increase in housing prices, many urbanites still have taken a loan from their bank or family member, even if they wind up as “fangnu”, or “house slaves”, a colloquial term for those who spend a substantial portion of their family income on a mortgage. It is a health hazard for the Chinese, though, to worry about housing payments in and of itself when housing prices increase irrationally. Then, is there some link between rapidly-rising housing prices and deteriorating physical health in China? This is the question that this study attempts to answer.

Existing studies investigating the effect of family wealth on economic behaviors reveal that housing price fluctuations can affect female labor force participation [1, 2], fertility and marriage rates [3–7], entrepreneurship [8], portfolio choice [9], education [10, 11], and consumption [12]. However, research on the effect of housing prices on physical health is relatively limited.

To the best of my knowledge, some studies have explored the impact of housing fluctuations on physical and mental health in developed countries, such as the UK, the US, and Australia, and mostly focus on a subset of homeowners or renters. However, there are four striking differences between China’s real estate market and that of other developed countries in terms of homeownership rate, housing affordability stress, housing loan, and preference for a house. First, China’s homeownership exceeded 70% between 2012 and 2018, which is higher than that in the UK, the US, and German. Second, the housing price-to-wage ratio released by the International Monetary Fund indicates that housing prices in China are severely out of proportion to residents’ income. Third, personal housing loans increased rapidly in China, accounting for the largest proportion of the total loan. Fourth, China has a traditional custom that regards a house as a prerequisite for a marriage. Thus, the impacts of housing prices on Chinese health status may be different from that in developed countries.

China has witnessed rapidly rising housing prices in past years, providing a unique research context. The aim of this study is to explore the effect of housing prices on individual physical health. First, we start the theoretical analysis by elaborating the dual effects of housing prices on individual physical health carefully, including both the positive wealth effect and negative “house slave effect”. Overall, the final net effect is complicated, as two different effects jointly influence individual health; however, I assume the final net effect is negative. Then, I empirically test the predictions using the Chinese General Social Survey (CGSS) data. Note that endogeneity issues may bias the results. Hence, I introduce the province’s land supply area as the instrument for housing prices and use the two-stage least square (2SLS) method to re-examine the relationships between housing prices and individual physical health. The empirical results support the predictions that rising housing prices adversely affect individual physical health. Finally, to gain further insights into the underlying channels, I investigate the effect of housing prices on subjective social status, physical exercise, and mental health.

This study contributes to several strands of literature. First, it contributes to the study of housing wealth and health. The existing literature only focused on a subset of homeowners or renters in developed countries, whether it be wealth effect or of economic barometer action. This study looks at the health effect of rising housing prices on a broader segment of the population in China. Importantly, this study finds that the “house slave effect” outweighs the wealth effect, and that an increase in housing prices adversely affects the physical health for a broader segment of the population. Second, considering China has a high homeownership and around 20% of metropolitan households own several residences [13], this study explores heterogeneous effects by number of houses, not house tenure status [14]. Third, this study complements recent research devoted to exploring the influence of social norm from an economics perspective. The social norm can be described as “a man should earn more than his wife; a woman should take care of the family” in some western countries [15–17]. These studies show that the social norm may induce an aversion to situations where the wife earns more than her husband, thereby affecting marital formation. This study reveals that China’s social norm of men purchasing a house pre marriage, accompanied by a sustained rise in housing prices, has led to the deterioration of men’s health status.

The remainder of the study is organized as follows. Section 2 proceeds with the literature review and hypotheses development. Data sources, the definition of variables, and summary statistics are presented in Section 3. Section 4 describes the empirical strategy. Section 5 provides the baseline results and discusses the identification issues. A series of robustness checks are shown in Section 6. Section 7 explores a few heterogeneous effects and possible mechanisms. The concluding remarks and policy implications are summarized in Section 8.

## Section 2: Literature review and hypotheses development

### 2.1. Factors affecting physical health

Research has explored the factors affecting the physical health. For instance, as documented by studies in health economics and medicine, lottery wins [18, 19], macroeconomic fluctuations [20], income shocks [21], work intensity [22, 23], and neighbourhood deprivation [24] are all associated with health status. Other studies also exploit the health effect of trade liberalization and pollution [25]. Unlike existing findings, this study underlines the role of housing prices fluctuations in influencing health problems.

### 2.2. The effects of housing prices on physical health

Although an extensive literature focuses on housing prices or health, the nexus between them in China is rarely considered. For example, some studies use the plausibly exogenous housing prices fluctuations in UK to show that an increase in housing prices positively affects homeowner’s mental wellbeing [26, 27]. However, one limitation of study by Ratcliffe (2015) [26] is that the author does not exclude whether economic conditions have played a role. [14] finds an increase in housing prices positively affects the physical health of outright owners, and negatively affects the physical and mental health of renters. Another strand of the literature is not consistent with the wealth effect. Joshi (2016) [28] finds housing prices act as an economic barometer, and an increase in housing prices can advantage both homeowners and renters. The literature has only focused on a subset of homeowners or renters in developed countries, whether it be on the wealth effect or of economic barometer action.

### 2.3. Hypotheses development

In theory, the Grossman health demand model reveals that that health is typically considered as a normal good (as well as consumption) which can be determined by many economic resources, such as income or wealth, as documented by Grossman (1972) [29]. Research shows that an increase in housing prices can promote consumption through the realized wealth, unrealized wealth and liquidity constraint effects for homeowners [30–32]. Likewise, the housing wealth effect also advantages health through similar ways. First, homeowners can “cash out” the wealth into real income, by replacing their house with a smaller value house or selling their houses. Second, even if house owners do not directly realize their real estate value, the unrealized wealth can bring irrational expectations and create the illusion of being wealthier. Finally, house owners can obtain more credit funds by mortgaging value-added real estate to banks to relax family liquidity constraints, supporting the collateral channel documented by James et al. (2019) [33]. As aforementioned, all three ways will somewhat increase the real income of house owners, thereby increasing health-related investments such as physical exercise and superior medical services. The input on health-related investment in turn advantages health.

However, the wealth effect in western countries cannot be completely applied to China due to the uniqueness of the Chinese property and financial markets because many constrains, such as sale and purchase restriction policies and collateral constrained mortgages, hinder homeowners from realizing the wealth effect by “cashing out”. Notably, the Chinese have a cultural custom of valuing a house. Furthermore, owning a house is usually a premise for marriage. BBC reported that 70% of Chinese millennials are homeowners and a sizeable 91% plan to buy a house in the next five years [34]. In particular, young people’s wages are too low to afford expensive housing prices; therefore, their families are expected to financially assist in the pursuit of a house. In sum, not only younger generations but also their parents must desperately work harder and compress consumption related to health investment for paying the house. After buying a house, they also suffer the heavy financial burden induced by the loan mortgage. This can be called the “house slave effect” in China. This “house slave effect” outweighs the wealth effects and definitely threatens health through different channels. Based on the this analysis, this study proposes the first hypothesis:

**Hypothesis 1: Housing prices have a significantly negative effect on physical health**.

How does the “house slave effect” due to high housing prices affect physical health? This can be explored via three channels. The first channel can be the subjective social status. A handful of studies demonstrate that a house is the symbol of economic, sociocultural, and psychosocial values, which influences the recognition of subjective social status [35]. Naturally, families who already own a house should enjoy the benefits from asset appreciation due to the housing wealth effect, and their level of subjective social status will increase accordingly. However, the wealth effect in western countries cannot be completely applied to China. In contrast, the “house slave effect” in China can not be ignored, that may lower individual subjective social status, especially for those with heavy house loan. For renters, when house prices increase, people tend to reduce consumption and work longer to realize their dream of owning a house, thereby lowering their social status [36]. In particular, even for the general public in China, most households only have one house, and they are difficult to cash out the wealth from the asset appreciation [37]. On the contrary, they must bear the inflation pressure caused by rising housing price. The increase in living cost will constrain their budgets and compress the consumption, naturally lowering their subjective social status [38]. In addition, some empirical studies focusing on other countries also postulated that the rise in housing prices might generate housing wealth inequality, which lowers the subjective social status [39–41].

Notably, the subjective social status affects individual health beyond the effects of objective social-economic status on health [42, 43]. On the one hand, socioeconomic status affects people’s working and living conditions, and determines their access to healthcare and high-quality services; on the other hand, socioeconomic status affects people’s psychological state and cognition of the outside world, such as self-esteem and self-efficacy [44, 45]. These material and psychological factors jointly influence people’s behavior and susceptibility to pathogens. Recent study reveals that subjective social status was associated with some diseases and other mortality [46]. Hence, rising housing prices may disadvantage individual health through lowered subjective social status.

Physical exercise may be another potential mechanism. As elaborated by the “house slave effect”, rising housing prices will compress consumption, including health-related investment such as physical exercise [47]. The Grossman health capital model proposed by Grossman (1972) states that physical exercise is an important input into health production process. Reduced physical exercise definitely worsens health status. Further to avoid the purchase and loan limit policy, and enjoy preferential policies, such as taxes, down payment ratio, and loan interest, some people get a marriage or fake divorce, which leads to significantly negative impacts on mental health. In addition, repayment pressure stemming from house loan adds to more mental stress. The causal relationship between mental health and some diseases, such as coronary heart diseases and clinical depression, and mortality, has been widely investigated by Strike and Steptoe (2004) [48]. They report that psychological stress is correlated with diseases and mortality. Therefore, I infer mental health plays an important role as the third channel. Based on the this analysis, this study proposes the second hypothesis:

**Hypothesis 2: Housing prices indirectly affect physical health through subjective social status, physical exercise, and mental health**.

## Section 3: Data, variables and summary statistics

### 3.1. CGSS and CHNS data

This study uses two macro datasets for the empirical analysis. The first dataset is the CGSS, which is administered by the National Survey Research Center at Renmin University of China. The samples for this survey are drawn using stratified, multistage, and Probability-Proportional-to-Size Sampling. It is implemented since 2003, once every one or two years, with the latest year being 2021. This study uses the three waves in 2012, 2013, and 2015 in the main results considering the questionnaires are consistent during the period. Further, I extended the data to 2021 in robustness checks to capture the period when the housing prices increased rapidly.

The CGSS is high-quality data of cross-section data which contains a wealth of information about demographics, income (individual and family), house property and marriage concept. It also covers rich information about individual health status, such as self-rated physical health, height and weight for the calculation of BM, which this study is interested in. Moreover, it also includes subjective social status, mental health status, and health-related behaviors for the mechanism analysis. However, a limitation of the CGSS survey data is that it only includes the interviewee’s provincial geographic information considering the privacy of respondents, thus making it impossible to match the CGSS data to city-level housing prices. To analyze the effect of city-level housing prices on individual health, this study introduces the second dataset: the China Health and Nutrition Survey.

The CHNS is initiated in 1989 by the Carolina Population Center at the University of North Carolina. Here, the city geographic information from 2000 to 2011 are directly obtained from Fan et al. (2020). The CHNS data are mainly pooled cross-section with a total of nine years, including 1989, 1991, 1993, 1997, 2000, 2004, 2006, 2009, 2011, and 2015. This study only uses the five waves ranging from 2000 to 2011. The choice for the sample can be explained by two reasons. First, the housing prices data is not available prior to 1998. Second, I cannot obtain the detailed city geography information in 2015 wave. The CHNS data covers a wealth of information about personal health, including illness and injury status and type of illness. This study uses the CGSS data in the main results and mechanism analyses, and the CHNS data are used for the robustness examinations.

### 3.2. Health outcome status

This study has two health status measures as dependent variables. The first health status measure is self-rated physical health, which is constructed according to the question “How do you feel about your current health status?”. Respondents are asked to select from a five-point scale option: very poor, poor, fair, good, excellent. The five options are assigned a number from 1 to 5, respectively. The large the number, the better the health status. The second health status measure is Body Mass Index (BMI). BMI is a widely accepted indicator for determining the degree of obesity, and the World Health Organization also utilizes BMI to define obesity or overweight. Obesity, in particular, has a detrimental effect on nearly every aspect of health, from reproductive and pulmonary function to cognition and mood. Simultaneously, obesity raises the risk of developing a variety of debilitating and fatal conditions, such as diabetes, heart disease, and some types of cancer. BMI somewhat reflects the real state of individual physical health. I calculate BMI by dividing respondent’s weight (kg) by the square of their height (m). According to the Chinese BMI standard, obesity is defined when BMI is more than 28. Following the BMI standard, I create a binary variable and assign it a value of 1 if the BMI is greater than 28, and otherwise 0.

### 3.3. Housing prices

Housing prices data at the province and city levels both come from the China premium database of CEIC. The data that are used in the basic results includes three waves of 2012, 2013, and 2015, covering 29 province in total. I adjust the housing prices to 2011 prices using the consumer prices index.

### 3.4. Instrumental variable

Following Binkai and Rudai (2013) [37], this study uses province-level land supply as an instrumental variable for housing prices in the IV regressions. Land is the primary input element in the housing market. On the one hand, land supply deficit may account for an increase in housing prices, satisfying the correlation requirements. On the other hand, the land supply area only can affect individual health via housing prices fluctuations, as land supply is strictly regulated by the central and provincial governments. Thus, I think they can serve as credible instrument for housing prices. The instrument data are obtained from the “Statistical Yearbook of Land and Resources of China”.

### 3.5. Control variables

According to prior literature [14, 27], individual and family-level characteristics have direct impacts on health. Hence, this study controls for them. Specially, these variables are as followed: 1) Individual income; 2) Age and age square; 3) Year of schooling; 4) Gender (female = 1, otherwise = 0); 5) Ethnicity (Han ethnicity = 1, otherwise = 0); 6) Residential status (living in urban area = 1, otherwise = 0); 7) Hukou (urban hukou = 1, otherwise = 0); 8) Migration status (not living in the hukou registration location = 1, otherwise = 0); 9) Occupation (full-time work but not peasant = 0, full-time peasant = 1, unemployed = 2);10) Family income; 11) Children (the number of participants’ children under 18 years). All individual and family characteristics are obtained from the CGSS database.

This paper also controls for other province-level variables, including GDP per capital, unemployment rate, population, transportation facilities, education, medical service, and PM2.5 (in natural log). All province-level variables except for PM2.5 come from China Premium Database of CEIC. The data with respect to PM2.5 are obtained from Atmospheric Composition.

### 3.6. Final sample

Finally, this study pooled the three waves of CGSS data and matched them to the province-level economic data. The final sample contains 34,090 individuals. The left panel of Table 1 displays summary statistics for the variables used in the empirical strategy for the full analysis sample. Subsample statistics for individuals with only one house and more than one house are presented in the middle and right panel, respectively. Observing Table 1, I find two facts. First, the average is 49 years old and the average self-rated physical health is 3.620 for the full sample. Second, the average self-rated physical and mental health for the subsample with one house are 3.593 and 3.849, smaller than the subsample with more than one house. This indicates that the self-rated physical health status is better for individuals with more houses. Table 2 shows the descriptive statistics for provincial characteristics respectively.

**Table 1:** Summary statistics for individual characteristics.

Panel A: Individual and family characteristics from CGSS data									
	Full sample			With one house			With more than one house		
	(1)	(2)	(3)	(4)	(5)	(6)	(7)	(8)	(9)
Variables	N	Mean	Std.Dev.	N	Mean	Std.Dev.	N	Mean	Std.Dev.
**Health measures**									
Self-rated physical health (1 to 5)	34,077	3.620	1.084	29,075	3.593	1.094	5,002	3.779	1.009
Mental health(1 to 5)	34,027	3.870	0.963	29,028	3.849	0.966	4,999	3.992	0.937
BMI	34,090	0.0629	0.243	29,085	0.0615	0.240	5,005	0.0709	0.257
**Demographics**									
Age	34,088	49.27	16.52	29,083	49.59	16.58	5,005	47.45	16.04
Revenue	31,167	8.447	3.312	26,608	8.360	3.309	4,559	8.954	3.286
Marriage status (1 married;0 otherwise)	34,067	0.105	0.307	29,068	0.102	0.303	4,999	0.124	0.330
Education	34,066	8.608	4.627	29,069	8.383	4.607	4,997	9.923	4.528
Gender (1 female; 0 otherwise)	34,090	0.505	0.500	29,085	0.511	0.500	5,005	0.469	0.499
Residencial status (1 urban; 0 otherwise)	34,090	0.601	0.490	29,085	0.588	0.492	5,005	0.679	0.467
Hukou (1 Urban; 0 otherwise)	34,090	0.449	0.497	29,085	0.435	0.496	5,005	0.531	0.499
Employment status	34,085	0.997	0.881	29,081	1.022	0.875	5,004	0.854	0.903
Ethnicity (1 Han Chinese; 0 otherwise)	34,050	0.917	0.276	29,049	0.916	0.277	5,001	0.923	0.267
Migration status	34,090	0.534	0.499	29,085	0.528	0.499	5,005	0.566	0.496
**Family characteristics**									
Family revenue	30,288	10.31	1.480	25,907	10.21	1.496	4,381	10.88	1.237
Number of children	29,663	0.472	0.734	25,399	0.471	0.737	4,264	0.483	0.717

**Table 2:** Summary statistics for province characteristics.

	(1)	(2)	(3)	(4)	(5)
Variables	N	Mean	Std.Dev.	Min	Max
Housing prices (Ten thousands of yuan per sq.meter)	34,090	0.641	0.407	0.338	2.101
GDP per capital (ten thousands of yuan)	34,090	5.085	2.241	1.971	10.80
Unemployment rate (%)	34,090	3.339	0.673	1.200	4.500
Population (ten thousands of people)	34,090	5,307	2,647	573	10,849
Education (schools per ten thousands of people)	34,090	12.42	2.681	7.479	19.83
Medical service(Hospitals per capital)	34,090	0.189	0.0592	0.100	0.374
Transport facility(Trams per ten thousands of people)	34,090	10.5	2.92	6.29	21.7
PM2.5 (in natural log)	34,090	41.56	16.10	13.10	82.59
Supply land (hectares)	34,090	4,266	3,024	245.7	14,407

## Section 4: Estimation method

To examine the effect of housing prices on health outcomes, this study employs the following benchmark empirical specification:

$$\begin{array}{c} {\text{Health}}_{ipt}=\alpha +\beta {HP}_{pt}+\gamma_{1}X_{ipt}^{\text{individual}}+\gamma_{2}X_{ipt}^{\text{family}}+\gamma_{3}X_{pt}^{\text{province}\;}+\mu_{p}+\lambda_{rt}+\varepsilon_{ipt} \end{array}$$ (1)

Among them, the subscripts i, p, and t represent the individual, province, and year, respectively. The dependent variable *Health*_*ipt*_ refers to the health status, including self-rated physical health and BMI. *HP*_*pt*_ is the housing price at the provincial level, which is the main variable of interest. The coefficient *β* can capture the effect of rising housing prices on health outcomes. I expect the estimated effect to be negative and statistically significant, indicating housing prices adversely affect individual health. This study adds to a set of individual and family-level covariates, $X_{ipt}^{\text{individual}\;}$ and $X_{ipt}^{\text{family}\;}$, respectively. For the preferred specification, this study includes all individual-level and family-level controls (see the note in Table 1 for full list of variables). In section 5.1, this study explores a series of different sets of controls before presenting the results for the preferred specification.

$X_{pt}^{\text{province}\;}$ is a comprehensive set of province-level controls for the benchmark specification. Some macroeconomic controls such as GDP per capital, population, transportation facilities, education, medical service, and unemployment rate affect housing prices and individual health simultaneously; hence, it is crucial to control for them. This study also controls for PM2.5 (in natural log) to capture the effect of air pollution on health. *μ*_*p*_ is the unobserved time-invariant province-fixed effects. According to Bombardini and Li (2020) [16], the region*year effects *λ*_*rt*_ are used in to capture for differential trends in health status changes across three regions in China. There are three regions: East (Beijing, Tianjin, Hebei, Shanghai, Jiangsu, Zhejiang, Fujian, Shandong, Guangdong and Hainan), Middle (Shanxi, Anhui, Jiangxi, Henan, Hubei and Hunan), West (Inner Mongolia, Guangxi, Chongqing, Sichuan, Guizhou, Yunnan, Xizang, Shanxi, Gansu, Qinghai, Ningxia and Xinjiang). In addition, the results are robust to include both the province-fixed effects and year-fixed effects (results showed in S1 Table). *ε*_*ipt*_ is the error term.

## Section 5: Results

### 5.1. Baseline results

Since the dependent variable is an ordered variable, this study chooses the ordered probit method for the benchmark regression. Table 3 shows the regression results. The dependent variable is self-rated physical health in column (1)-(3). The last three columns present results when BMI is the dependent variable. This alternative indicator reflects respondents’ actual physical health, remedying the subjectivity of self-rated physical health.

**Table 3:** Basic results for the effect of housing prices on individual physical health.

Dependent Variable	Self-rated physical health			BMI		
	(1)	(2)	(3)	(4)	(5)	(6)
Housing prices	-0.226**	-0.255**	-0.461***	0.353**	0.317	0.547**
	(0.088)	(0.102)	(0.138)	(0.169)	(0.196)	(0.258)
Revenue		0.019***	0.018***		0.012**	0.012**
		(0.003)	(0.003)		(0.005)	(0.005)
Female		-0.108***	-0.109***		-0.030	-0.031
		(0.015)	(0.015)		(0.026)	(0.026)
Age		-0.051***	-0.051***		0.027***	0.027***
		(0.003)	(0.003)		(0.006)	(0.006)
Age square		0.000***	0.000***		-0.000***	-0.000***
		(0.000)	(0.000)		(0.000)	(0.000)
Urban hukou		-0.015	-0.015		0.117***	0.119***
		(0.020)	(0.020)		(0.036)	(0.036)
Urban		0.054***	0.055***		0.061	0.060
		(0.020)	(0.020)		(0.037)	(0.037)
Education		0.015***	0.015***		-0.018***	-0.018***
		(0.002)	(0.002)		(0.004)	(0.004)
Han		-0.078**	-0.078**		-0.109**	-0.108**
		(0.031)	(0.031)		(0.052)	(0.052)
Migration		-0.022	-0.020		0.032	0.031
		(0.019)	(0.019)		(0.034)	(0.034)
Unmarried		0.036	0.041		-0.149	-0.144
		(0.079)	(0.079)		(0.154)	(0.155)
Employment(ref. = Employed in non-agricultural work)						
Employed in agricultural		-0.148***	-0.147***		-0.012	-0.012
		(0.023)	(0.023)		(0.043)	(0.043)
Unemployed		-0.262***	-0.262***		0.114***	0.113***
		(0.020)	(0.020)		(0.036)	(0.036)
Revenue		0.019***	0.018***		0.012**	0.012**
		(0.003)	(0.003)		(0.005)	(0.005)
Family revenue		0.078***	0.078***		0.016	0.016
		(0.006)	(0.006)		(0.010)	(0.010)
Number of children(age<18)		0.031**	0.031**		-0.004	-0.004
		(0.013)	(0.013)		(0.024)	(0.024)
Province controls	NO	NO	YES	NO	NO	YES
Province fixed effects	YES	YES	YES	YES	YES	YES
Region*Year fixed effects	YES	YES	YES	YES	YES	YES
Observations	34,077	25,773	25,773	34,090	25,782	25,782
Pseudo R-squared	0.0137	0.0791	0.0793	0.0231	0.0323	0.0330

Notes: All regressions include province fixed effects and Region*Year fixed effects. Columns (1) and (4) only control for housing prices, and Columns (2) and (5) add individual and family characteristics. Columns (3) and (4) further add province charactertistics. To save space, the province charactersitics controls are not reported, including GDP per capital, unemployment rate, population, transportation facilities, education, medical service and PM2.5 (in natrual log). The constant term is omitted. Robust stand errors are in parenthesis.

*** p<0.01,

** p<0.05,

* p<0.1.

The variable of interest is housing price. The coefficient on this variable captures the effect of housing price on physical health. All columns of Table 3 control for province fixed effects and region*year fixed effects. Columns (1) and (4) only control for housing prices, and Columns (2) and (5) add individual and family characteristics. Columns (3) and (6) present the regression results from the preferred specification, including province-level variables, such as unemployment rate.

Overall, the baseline results support the predictions. As displayed by the first three columns, the estimated coefficients on housing prices are negative and statistically significant, suggesting that rising housing prices are associated with a significantly higher likelihood of suffering from self-rated physical health problems. The coefficient on the housing price in column (5) is positive but not significant. However, when controlling for the province controls in column (6), the estimated effect is positive and statistically significant at 1% level, which means that rising housing prices lead to more obesity problems. Thus, Hypothesis 1 is supported.

As for control variables, the estimated result in column (3) is consistent with theoretical expectations and empirical study. First, increases in individual and family income significantly improve self-rated physical health. The finding is consistent with the fact that as one of the most important economic factors, income improves investment in health. Second, education has a positive effect on self-rated physical health. Third, respondents in urban areas are more likely to self-rate themselves to be physical healthy, which is consistent with McDade and Adair (2001) [49]. One possible explanation is that urban cities have better public services, high-quality education, and medical resources. In addition, urban cities provide diverse employment opportunities and salaries that are more competitive. Therefore, urban residents have higher expenditure on health investment, and thus, self-rated physical health is higher. Finally, this study finds that female are more likely to be stuck in bad physical health status.

### 5.2. Identification concerns

The baseline results are in favor of the initial hypothesis, relying on the assumption that housing price is uncorrelated with the error term. One particular concern for the assumption is whether the empirical strategy omits some unobservable factors correlated with housing price and health status simultaneously, which can lead to biased coefficient estimate. For example, local economic growth will raise local housing prices. Meanwhile, economic growth is accompanied by environmental pollution, which negatively affects individual health. In addition, if labor can move freely between different cities, it will also choose a city suitable for them based on housing prices and health conditions. These factors are correlated with both housing price and health, but it is difficult to measure them. To address the endogeneity issue, this study introduces land supply at the province-level as an instrumental variable for housing price.

In Table 4, this paper re-estimates the regression using the two-stage least squares (2SLS) methodology. To save space, only the estimated coefficient on the key variable are presented here. The first-stage results are displayed in the first half of Table 4. The values of Kleibergen-Paap Wald rk F and Cragg-Donald Wald F statistics are far greater than the critical value of 10, indicating no concerns of weak instrumental variables. In addition, as expected, all instruments are statistically significant and have negative sign in the first stage results. The second-stage results are reported in the second half of Table 4. Columns (1) and (2) present the estimation results when the dependent variables are self-rated physical health and BMI, respectively. The estimated coefficient on housing price in column (1) is still negative and statistically significant, consistent with the result in column (3) of Table 3. Further, the estimated effect in column (2) of Table 4 shows that an increase in housing price is correlated with a larger BMI.

**Table 4:** The 2SLS results for the effect of housing prices on individual physical health.

	(1)	(2)	(3)	(4)
	2SLS	2SLS	2SLS	2SLS
**The first stage**				
**Dependent Variable**	**housing prices**	**housing prices**	**Ln(housing prices)**	**Ln(housing prices)**
Supply land	-0.027***	-0.027***	-0.018***	-0.018***
	(0.001)	(0.001)	(0.000)	(0.000)
Cragg-Donald Wald F statistic	3657.768	3655.559	1152.868	1152.065
K-P Wald rk F	3184.751	3655.559	1189.288	1188.342
**The second stage**				
**Dependent Variable**	**Self-rated physical health**	**BMI**	**Self-rated physical health**	**BMI**
Housing prices	-1.025***	0.264**		
	(0.355)	(0.103)		
Ln(housing prices)			-2.331***	0.600**
			(0.808)	(0.234)
Observations	25,773	25,782	25,773	25,782
R-squared	0.205	0.015	0.200	0.011
Province characteristics	YES	YES	YES	YES
City characteristics	YES	YES	YES	YES
Province FE	YES	YES	YES	YES
Region*Year FE	YES	YES	YES	YES

Notes: The table reports the 2SLS estimation results.he controls in all columns are the same as column (3) of Table 3. The constant term is omitted. Robust stand errors are in parenthesis.

*** p<0.01,

** p<0.05,

* p<0.1.

In columns (3) and (4), this study adopts the logarithm of housing price as the key independent variable. The estimated effect remains negative and statistically significant at the 1% level in column (3). Notably, the estimated effect is also quantitatively sizable. In particular, the result in column (3) suggests that a 10% increase in housing price is associated with a 0.2331-scale decrease in self-rated physical health. Observing column (4), a 10% increase in housing price is associated with 0.06 increase in BMI. The estimation results in columns (3) and (4) provide clear evidence that individuals stuck in rising housing prices are more likely to be self-rate themselves as physical unhealthy and have higher BMI.

## Section 6: Additional robustness checks

Next, I perform a variety of robustness checks on the estimation results. First, I check their sensitivity to alternative health status and housing prices. Second, I adopt the most stringent standard errors to ensure the robustness of the finding. Third, the CHNS data are employed with city geographic information and check whether the results are sensitive to city-level housing prices. Finally, a longer sample period is considered.

### 6.1. Alternative measurement of important variables

The main dependent variable is the self-rated score measuring the physical health status, ranging from one (the least health) to five (the most health). Respondents were asked to answer the question: “How do you feel about your present health status?”. Responses are on a five-point scale options: “very poor”, “poor”, “fair”, “good” and “excellent”, which are assigned an number from 1 to 5, respectively. To check whether the finding is robust to alternative dependent variable, this study first converts the five-point category variable into three-level category variable named self-rated physical health_1. Specifically, “very poor” and “poor” are classified as unhealthy with the value of 0, “fair” as neutral with the value of 1, and “good” and “excellent” as healthy with the value of 2. Second, this study generates a dummy defined as self-rated physical health_2, which equals 1 if the health status is excellent / good / fair, and 0 otherwise. Next, there is a question “How often has your work or other daily activities been affected by health issues in the past four weeks?”. The respondents are asked to provide a self-rated number on the frequency from 1 (the most) to 5 (the least). Accordingly, this study constructs the third health measure named self-rated physical health_3. The results are reported in Table 5. The estimated coefficients of housing prices are negative and statistically significant, consistent with the baseline regression. These findings continue to support the predictions that the increase in housing prices significantly diminish the physical health status.

**Table 5:** Alternative for the health status measurement and housing prices.

	(1)	(2)	(3)	(4)	(5)
Dependent Variable	health_1	health_2	health_1	Self-rated physical health	Self-rated physical health
Housing prices	-0.625***	-0.537**	-0.310**		
	(0.157)	(0.214)	(0.149)		
Commercial housing prices				-0.416***	
				(0.129)	
Average housing prices growth					-1.248***
					(0.280)
Individual controls	YES	YES	YES	YES	YES
Family controls	YES	YES	YES	YES	YES
Province controls	YES	YES	YES	YES	YES
Province fixed effects	YES	YES	YES	YES	YES
Region*Year fixed effects	YES	YES	YES	YES	YES
Observations	25,731	25,731	25,671	25,731	25,731
Pseudo R-squared	0.101	0.162	0.072	0.080	0.080

Notes: The controls in all columns are the same as column (3) of Table 3. The constant term is omitted. Robust stand errors are in parenthesis.

*** p<0.01,

** p<0.05,

* p<0.1.

Next, this study substitutes housing prices with two alternative measures as follows. The first is commercial housing prices. Apart from living in residential houses, individuals may live in urban villages, ordinary commercial apartments and high-end apartments. Due to the lack of data on the transaction prices of informal houses, commercial housing prices can be a reasonable alternative indicator. The past increase in housing prices may form people’s expectations on the increasing trend in future, and such expectations can affect the current health of the individual. Hence, the second alternative is average annual growth rate of housing prices in the past three years. Column (4) and (5) in Table 5 present the results with the two alternative housing prices as independent variables. This results indicate that rising housing prices continue to have detrimental and statistically significant effects on self-rated physical health.

### 6.2. Database change

As mentioned earlier, I cannot match individual health information to the city-level housing price as the hiding of interviewee’s city geographic information is anonymized in CGSS data. Consequently, I can just identify the causal relationship between provincial housing prices and health in the baseline regression. Actually, the provincial housing price may not necessarily be the real housing price level faced by the interviewee, since the real housing prices across cities within the same province may vary greatly. To address these issues, this study re-estimates the baseline empirical equation using the CHNS database. The estimation results are presented in Table 6. In all columns, the independent variables are the city-level housing price (in natural log). In the first column (1), the explained variable is a binary variable that equals 1 if an individual has experienced illness or injury in the past four weeks, and 0 otherwise. Column (1) draws a full sample for the regression. This results find that rising housing price significantly increases the chance of illness or injury. In Columns (2)-(6), I restrict the sample to individuals with occupations related to factory workers. Column (2) presents the estimation result with the same dependent variable as column (1). Next, to examine the effect of housing price on different types of diseases, I also construct five binary variables as dependent variables in Columns (3)-(6). For example, fever is a binary indicator that equals 1 if an individual suffered from one of the two injures, and 0 otherwise. As results displayed in columns (3)-(6), the estimated effects of housing prices on the specific types of illness including fever, muscle aches, and stomach pain are also statistically significant and negative.

**Table 6:** Change of the database.

	Using all occupations	Using occupations related to factory workers				
	(1)	(2)	(3)	(4)	(5)	(6)
Dependent Variable	Injury or illness	Injury or illness	Incomfortable or exhaustion	Fever,score throat,cough	Muscle pain and fracture	Stomachache,asthma
Ln(housing prices)	0.103***	0.175***	0.056**	0.090**	0.062***	0.037**
	(0.037)	(0.049)	(0.025)	(0.036)	(0.022)	(0.016)
Control variables	YES	YES	YES	YES	YES	YES
Occupation type dummies	NO	YES	YES	YES	YES	YES
Employer ownership dummies	YES	YES	YES	YES	YES	YES
Year fixed effects	YES	YES	YES	YES	YES	YES
Province fixed effects	YES	YES	YES	YES	YES	YES
Observations	5,171	1,635	1,636	1,636	1,636	1,636
R-squared	0.096	0.099	0.062	0.057	0.049	0.046

Notes: The included individual controls are: Marriage, gender, education, yearly-income, age age-square residential status, disease history, smoking behavior, health insurance, house value and house size. The city-level controls are: Productivity, education, education, urbanization, air quality index and water pollution. The policy shocks are: Export policy uncertainty and quota policy. The constant term is omitted. Robust stand errors are in parenthesis.

*** p<0.01,

** p<0.05,

* p<0.1.

### 6.3. Alternative sample period

According to National Bureau of Statistics, the national housing prices increased by 96% from 2010 to 2021. To capture the period when the housing prices increased rapidly, I assessed whether the results are sensitive to a longer period of time by supplementing new data in 2011, 2017, 2018, and 2021. Table 7 presents the estimation results applying this new sample period. In the first two columns, the independent variables are housing prices. In column (1), the estimated coefficient of housing prices is negative and statistically significant, indicating the detrimental effects of housing prices on self-rated physical health. In column (2), the estimated effect is positive and statistically significant at the 1% level, indicating that rising housing prices lead to more obesity problems. As columns (1) and (2) in Table 7 show, the results remain robust in this new sample including more periods. The longer and updated data could capture physical health variation, strengthening the robustness of the findings.

**Table 7:** Alternative sample period.

	(1)	(2)
Dependent Variable	Self-rated physical health	BMI
Housing prices	-0.049**	0.201***
	(0.024)	(0.042)
Individual controls	YES	YES
Family controls	YES	YES
Province controls	YES	YES
Province fixed effects	YES	YES
Region*Year fixed effects	YES	YES
Observations	53,842	53,842
Pseudo R-squared	0,079	0.033

Notes: The included individual controls are: Marriage, gender, education, yearly-income, age age-square residential status, disease history, smoking behavior, health insurance, house value and house size. The city-level controls are: Productivity, education, education, urbanization, air quality index and water pollution. The policy shocks are: Export policy uncertainty and quota policy. The constant term is omitted. Robust stand errors are in parenthesis.

*** p<0.01,

** p<0.05,

* p<0.1.

## Section 7: Heterogeneity and mechanism analyses

Here, I explore heterogeneous effects across the number of houses that individuals own. First, I look at whether the health effect varies across individuals, especially, males versus females, and the young versus old. Then, I examine the potential mechanisms by which housing prices affect self-rated physical health.

### 7.1. Heterogeneous effects by the number of houses

Housing-price changes could affect physical health through a mix of the positive wealth effect and negative “house-slave effects”. These effects can differ by the number of houses. Some existing literature documents that housing prices have heterogeneous effects on labor supply, consumption, and martial stability for renters and owners. Regarding health effects, some studies also find different outcomes across tenure status. However, as discussed in the Introduction, China has a high homeownership, approximately reaching 90%. On top of this, more than 20% of urban households own more than one home. Hence, it is meaningful to explore the heterogeneous effects across the number of houses rather than house tenure status.

The theoretical rationale for heterogeneous effect is that housing prices jointly affect different groups (with one house or more than one house) through both the wealth effect and “house slave effect” in different ways. First, I consider the effect of rising housing prices on the group with one house. Rising housing prices increase more health-related investments, thus improving the individual health potentially. However, an up-movement in housing prices also leads to the “house slave effect” as displayed in Section 2, threatening individual health. Notably, for individuals with one house residing in their house for a long time, the increase in housing prices may not mean an increase in real wealth and should not affect health, which is consistent with Campbell and Cocco (2007) [12]. It is not difficult to understand that the negative “house slave effects” outweighs the positive wealth effect, and eventually lead to harmful health problems. Now, this study considers the effect of housing prices on the group with more than one house. An increase in housing prices leads to the positive wealth effect but no “house slave effect”. However, it is not easy for households to “cash out” the wealth gain into health-related investments, considering many constrains (such as sale and purchase restriction policies) and collateral constrained mortgages in China. Therefore, the final net effect of housing prices on group with more than one house is theoretically ambiguous.

To examine the assumption, this study divides the full sample into two subsamples in Table 8. In columns (1) and (2), this study focused on the first subsample with one house. In columns (3) and (4), this study utilizes the second subsample with more than one house. In all columns of Table 8, the estimated effects of housing prices on individual health are negative, but only significant in the first two columns (subsample with one house). Quantitatively, the estimated effects in column (1) and (2) are larger. Overall, the results displayed by Table 8 provide strong evidence supporting the assumption that the adverse effects on physical health are pronounced in the group with one house, and no significant negative impacts are observed for the sample with more than one house.

**Table 8:** Heterogeneous effects with respect to number of houses.

	With one house		With more than one house	
	(1)	(2)	(3)	(4)
Dependent Variable	Self-rated physical health	BMI	Self-rated physical health	BMI
Housing prices	-1.247***	0.252**	-0.409	0.166
	(0.417)	(0.119)	(0.787)	(0.251)
Individual controls	YES	YES	YES	YES
Family controls	YES	YES	YES	YES
Province controls	YES	YES	YES	YES
Province fixed effects	YES	YES	YES	YES
Region*Year fixed effects	YES	YES	YES	YES
Individual controls	YES	YES	YES	YES
Observations	20,633	20,638	3,665	3,666
R-squared	0.206	0.017	0.194	0.023
Cragg-Donald Wald F statistic	2845.765	2846.400	527.058	523.926
K-P Wald rk F	2479.873	2480.003	436.459	433.480

Notes: The controls in all columns are the same as column (3) of Table 3. The constant term is omitted. Robust stand errors are in parenthesis.

*** p<0.01,

** p<0.05,

* p<0.1.

### 7.2. Heterogeneous effects by age

Section 7.1 clearly show that an increase in housing prices has more adverse effect on the group with one house. Then, for the population with one house, do housing prices also have heterogeneous effects across age? The standard life-cycle model predicts that the elderly respond more strongly to unanticipated wealth shocks because they have fewer years to live. Research documents that an increase in housing-price has heterogeneous effects on consumption or labor supply that are correlated with life-cycle characteristics [50]. Considering this, this study also investigates whether there are life-cycle effects of housing prices on physical health across age groups in China. Note that the minimum legal age is 16 years old in China. Hence, this study sets the lower bound of young generation at the age of 16. The upper bound of young generation is set at the age of 45. Finally, I define young generation as individuals ranging from 16 to 45 and elderly group as individuals aged 45 and above. Table 9 presents the estimation results. The estimated effect is only statistically significant in young groups. The findings can be attributed to the life-cycle characteristics of housing wealth. The elderly tend to have more wealth than the younger generation when housing prices rise rapidly, thereby offsetting the negative “house slave effect”. Understandably, the rising housing prices have adverse health effects on the younger cohort but not the older one.

**Table 9:** Heterogeneous effects with respect to age.

	Ages 20 to 45		Ages above 45	
	(1)	(2)	(3)	(4)
Dependent Variable	Self-rated physical health	BMI	Self-rated physical health	BMI
Housing prices	-2.488***	0.604**	-0.790	0.109
	(0.860)	(0.277)	(0.495)	(0.132)
Individual controls	YES	YES	YES	YES
Family controls	YES	YES	YES	YES
Province controls	YES	YES	YES	YES
Province fixed effects	YES	YES	YES	YES
Region*Year fixed effects	YES	YES	YES	YES
Individual controls	YES	YES	YES	YES
Observations	7,187	7,189	13,446	13,449
R-squared	0.113	0.018	0.146	0.023
Cragg-Donald Wald F statistic	695.303	695.260	2048.679	2049.379
K-P Wald rk F	381.269	381.203	1993.149	1993.583

Notes: The controls in all columns are the same as column (3) of Table 3. The constant term is omitted. Robust stand errors are in parenthesis.

*** p<0.01,

** p<0.05,

* p<0.1.

### 7.3. Heterogeneous effects by gender

Females and males are known to differ in a battery of economic preferences [51]. Thus, it is interesting to examine whether the effect of housing prices on health differs by gender. For this purpose, this study divides the subsample into male and female subsamples in columns (1) and (2) of Table 10, respectively. The estimated effect in each subsample is negative and statistically significant. In column (3), this study employs the full sample and adds the interaction term of housing price with gender as supplementary regressor in Table 10. The results indicate that the estimated coefficient on housing price is significantly negative but that on the interaction term with gender is significantly positive, indicating that housing price has heterogeneous effects across gender. The male are more sensitive to the rising housing price. The difference across gender could be attributed to the marriage norm that men take the foremost responsibilities for buying a house. Just like study by Wei et al. (2017) [52], 71% of unmarried females prefer their future husband to own at least a house. Interestingly, only 48% of males hope their future wife to own one or more houses. This means that males are expected to offer a house that serves as one of the important premarital costs.

**Table 10:** Heterogeneous effects with respect to gender.

	Male	Female	Full sample	Female	Male
	(1)	(2)	(3)	(4)	(5)
Dependent Variable	Self-rated physical health	Self-rated physical health	Self-rated physical health	Marriage concept	Marriage concept
Housing prices	-0.848***	-0.531**	-0.736***	1.709**	2.905***
	(0.214)	(0.234)	(0.159)	(0.731)	(0.870)
Female			-0.176***		
			(0.029)		
Female*Housing prices			0.117***		
			(0.036)		
Observations	10,555	10,078	20,633	10,488	10,027
Individual controls	YES	YES	YES	YES	YES
Family controls	YES	YES	YES	YES	YES
Province controls	YES	YES	YES	YES	YES
Province fixed effects	YES	YES	YES	YES	YES
Region*Year fixed effects	YES	YES	YES	YES	YES
Endogeneity test				9.40***	15.27***

Notes: The controls in all columns are the same as column (3) of Table 3. The constant term is omitted. Robust stand errors are in parenthesis.

*** p<0.01,

** p<0.05,

* p<0.1.

To further prove the social norm of men expected to buy a house before marriage, this study introduces a question whether an individual agrees that a good job is worse than a good marriage, which proxies the social norm. Specifically, I generate a binary indicator that equals 1 if the answer is completely agree/relatively agree, and assigned the value of 0 if the answer is neutral / relatively disagree / completely disagree. Columns (4) and (5) in Table 10 report the results. The estimated coefficients of housing prices are positive and statistically significant for females and males. This indicates that as housing prices rise gradually, along with greater uncertainty, the expectation of men purchasing a house before marriage is amplified. In particular, the magnitude is larger for males than females, suggesting that males are more agreeable with the marriage concept that “a good job is worse than a good marriage”. In other words, males are more fiercely affected by the social norm than females. Naturally, males take more responsibilities for affording the house for marriage, leading to more serious health problems.

### 7.4. Mechanism analysis

Why do housing prices adversely affect individual physical health? Theory predicts that the “house slave effect” affects individual health through some potential channels, which are tested here. I divide the sample into two subsamples. The first three columns are the group owning one house. The last three columns are the other group owning more than one house.

As displayed in Section 2, individuals are usually stuck in a lower social status if they face rising housing prices. Undoubtedly, low subjective social status have adverse effects on individual health. In addition, under the “house slave effect”, people increase work intensity at the expense of physical exercise, thus suffering from serious psychological stress. Physical exercise and psychological stress are known to be closely to health and some diseases [53].

To examine the mechanisms by which housing prices affect individual health, this study introduces three variables: (i) subjective social status: a self-rated number ranging from 1 (the lowest) to 10 (the highest); (ii) physical exercise frequency: an ordered variable assigned an number ranging from 1 (the least time) to 5 (the most time); (iii) mental health: an ordered variable assigned a number ranging from 1 (the worst) to 5 (the best). The three variables were constructed based on the following questions. The first question was “What level do you think you are currently in?”. Respondents were asked to provide numerical response between 1 to 10, with 1 for the lowest social status and 10 for the highest social status. The second question was “In the past year, did you often engage in the following activities in your free time–participate in physical exercise?”. The five answers “never”, “several times a year or less” and “several times a month”, “several times a week” were assigned a number from 1 to 5, respectively. The third question was “In the past four weeks, how often did you feel depressed or depressed?”. The five answers “always”, “often”, “sometimes”, and “rare”, “never” were assigned a number from 1 to 5, respectively. These variables are used as dependent variables and Eq (1) is re-estimated using two-stage least square estimation method.

In column (1) and (2) of Table 11, the dependent variables are physical exercise frequency and mental health, respectively. The coefficients on the housing prices are negative and statistically significant at the 1% level, suggesting that rising housing prices are associated with physical exercise frequency and mental health. Column (3) of Table 11 displays the result when the dependent variable is subjective social status. The coefficient of housing price is negative and statistically significant at the 1% level. This indicates that an increase in housing prices causes a lower subjective social status. However, for the subsample with more than one house, the estimated effects are not statistically significant. Overall, the results imply that the effects of housing prices on subjective social status, physical exercise, and mental health are the channels through that housing prices increase the possibility of individuals being unhealthy. Thus, Hypothesis 2 is supported.

**Table 11:** Potential mechanisms.

	With one house			With more than one house		
	(1)	(2)	(3)	(4)	(5)	(6)
Dependent Variable	Exercise	Mental health	Subjective social status	Exercise	Mental health	Subjective social status
Housing prices	-2.123***	-1.650***	-1.982***	0.689	0.086	-1.090
	(0.561)	(0.391)	(0.719)	(1.199)	(0.756)	(1.335)
Individual controls	YES	YES	YES	YES	YES	YES
Family controls	YES	YES	YES	YES	YES	YES
Province controls	YES	YES	YES	YES	YES	YES
Province fixed effects	YES	YES	YES	YES	YES	YES
Region*Year fixed effects	YES	YES	YES	YES	YES	YES
Observations	20,592	20,607	20,551	3,658	3,664	3,651
R-squared	0.217	0.096	0.083	0.233	0.082	0.107
Cragg-Donald Wald F statistic	2848.711	2844.140	2804.175	524.820	523.986	523.003
K-P Wald rk F	2485.227	2472.746	2397.146	433.257	433.209	434.343

Notes: The controls in all columns are the same as column (3) of Table 3. The constant term is omitted. Robust stand errors are in parenthesis.

*** p<0.01,

** p<0.05,

* p<0.1.

## Section 8: Conclusions and policy implications

### 8.1. Conclusions

The significant increase in housing prices in China have undoubtedly generated public concerns regarding their effects on individual physical health. An increasing number of people are stuck in sub-optimal health status because of pressure, depression, fatigue, and low self-esteem emotions induced by buying a house. This study tests the causal relationship between rising housing prices and individual physical health based on the China General Social Survey data. The results imply that individuals with greater exposure to high housing prices are more likely to be unhealthy. The findings still hold even after conducting several robustness tests. Considering the potential endogeneity issues, this study re-examines the relationship using land supply as instrument for housing price. Results reveal, once again, an increase in housing-price adversely affects individual physical health. Heterogeneity analysis across the number of houses, gender, and age shows that that the adverse effects of housing prices are stronger on the sample with one house, and among the male and young generation (aged between 16 and 45).

### 8.2. Policy implications

The results also have important policy implications. The China’s real estate has experienced tremendous prosperity during the past ten years along with the economic growth. However, sky-rocking housing prices undeniably lead to negative health outcomes, which policymakers may have previously ignored. While the analysis focuses on the implications for China, I believe that the adverse health effects may emerge in other Asian countries with similar patterns of rising housing prices and underlying social norms. To alleviate the negative health effects, the government should control the housing price growth rate by using housing purchase restriction policy or loan restriction, while simultaneously supporting government-subsidized housing projects. In particular, for males, the negative health effects are stronger relative to the females because they are expected to buy a house before marriage. Hence, the government should gradually promote gender equality.

## Supporting information

S1 Table Results for the effect of house prices on individual physical health. (PDF)
