# Supplementary material for: Do housing prices affect individual physical health? Evidence from China
Source: PLoS One. 2024 Apr 17;19(4):e0299561. doi: 10.1371/journal.pone.0299561 (PMC11023592; doi:10.1371/journal.pone.0299561)
Supplement: S1 Table — (PDF) [file pone.0299561.s001.pdf]

Table 1. Results for the effect of house prices on individual physical health

	Full sample		With one house		With more than one house	
**Dependent Variable**	**(1)** **Self-rated physical health**	**(2)** **BMI**	**(3)** **Self-rated physical health**	**(4)** **BMI**	**(5)** **Self-rated physical health**	**(6)** **BMI**
House prices	-0.345***	0.553**	-0.521***	0.614**	-0.043	0.135
	(0.129)	(0.240)	(0.148)	(0.278)	(0.310)	(0.556)
Observations	25,773	25,782	20,633	20,638	3,665	3,666
Individual controls	YES	YES	YES	YES	YES	YES
Family controls	YES	YES	YES	YES	YES	YES
Province controls	YES	YES	YES	YES	YES	YES
Province fixed effects	YES	YES	YES	YES	YES	YES
Year fixed effects	YES	YES	YES	YES	YES	YES

Notes: The controls in all columns are the same as column (3) of Table 2. The constant term is omitted. Robust stand errors are in parenthesis. *** p<0.01, ** p<0.05, * p<0.1.
